# Mapping risks associated with soil copper contamination using availability and bio-availability proxies at the European scale

**DOI:** 10.1007/s11356-022-23046-0

**Published:** 2022-10-15

**Authors:** Laura Sereni, Bertrand Guenet, Isabelle Lamy

**Affiliations:** 1 UMR 1402 ECOSYS, Ecotoxicology Team, Université Paris-Saclay, INRAE, 78026 Versailles, AgroParisTech France; 2 Laboratoire de Géologie de L’ENS, UMR 8538, PSL Research University, CNRS, IPSL, Paris, France

**Keywords:** Trace element, Mobility, Transfer function, Risk assessment, Diffuse contamination, Regional assessment

## Abstract

Soil contamination by trace elements like copper (Cu) can affect soil functioning. Environmental policies with guidelines and soil survey measurements still refer to the total content of Cu in soils. However, Cu content in soil solution or free Cu content have been shown to be better proxies of risks of Cu mobility or (bio-)availability for soil organisms. Several empirical equations have been defined at the local scale to predict the amount of Cu in soil solution based on both total soil Cu content and main soil parameters involved in the soil/solution partitioning. Nevertheless, despite the relevance for risk assessment, these equations are not applied at a large spatial scale due to difficulties to perform changes from local to regional. To progress in this challenge, we collected several empirical equations from literature and selected those allowing estimation of the amount of Cu in solution, used as a proxy of available Cu, from the knowledge of both total soil Cu content and soil parameters. We did the same for the estimation of free Cu in solution, used as a proxy of bio-available Cu. These equations were used to provide European maps of (bio-)available Cu based on the one of total soil Cu over Europe. Results allowed comparing the maps of available and bio-available Cu at the European scale. This was done with respective median values of each form of Cu to identify specific areas of risks linked to these two proxies. Higher discrepancies were highlighted between the map of bio-available Cu and the map of soil total Cu compared to the Cu available map. Such results can be used to assess environmental-related issues for land use planning.

**Supplementary Information:**

The online version contains supplementary material available at 10.1007/s11356-022-23046-0.

## Introduction

From a spatial point of view, native indigenous trace elements in soils largely vary around the world due to bedrock. In addition, atmospheric deposition, agriculture, mine tailing, or industrial activities can be important exogenous sources of soil trace element contamination (Hong et al. 1996; Nicholson et al. 2003). Fluxes of trace elements in ecosystems include their accumulation in surface soil horizons and their release to the soil solution, to the organisms or until the aquifers. While trace elements are often required for biological systems, large amount may have toxic effects (Flemming and Trevors 1989; Shabbir et al. 2020). Among the trace elements, copper (Cu) is widely used in industrial and agricultural sectors. In the absence of contamination, Cu is found as a native trace element at various total concentrations in soils, typically from 5 until 50 mg. kg^−1^ of Cu depending on the bedrock, but concentrations above 100 mg. kg^−1^ of Cu can be observed in Australia or in Baltic shield (Salminen and Gregorauskiene 2000). Additionally, inputs from different sources like manure, pesticides, or fertilizers are regularly added, leading at a spatial scale to various total soil content at least in the surface soil horizon. The annual amount of Cu deposited on soils through atmospheric contamination or for agronomical purposes were estimated around 3900 gCu. km^−2^ year^−1^ (~ 0.01 mg. kg^−1^ of Cu) for atmospheric deposition and between 100 and 800 g. km^−2^ year^−1^ of Cu (respectively 0.003 and 0.025 mg. kg^−1^ of Cu) for agricultural inputs depending on the fertilizers and crop type (Azimi et al. 2004). Outputs by crops or leaching waters are more difficult to estimate (Romkens et al. 2004), but globally general pattern leads to Cu accumulation in surface horizons. Thus, most of the environmental quality standard are defined on the basis of the total soil metal content in the surface horizon, while the relevance of such a value in terms of risks for metal mobility or bio-availability had been questioned (Kördel et al. 2013).

**Fig. 1:**
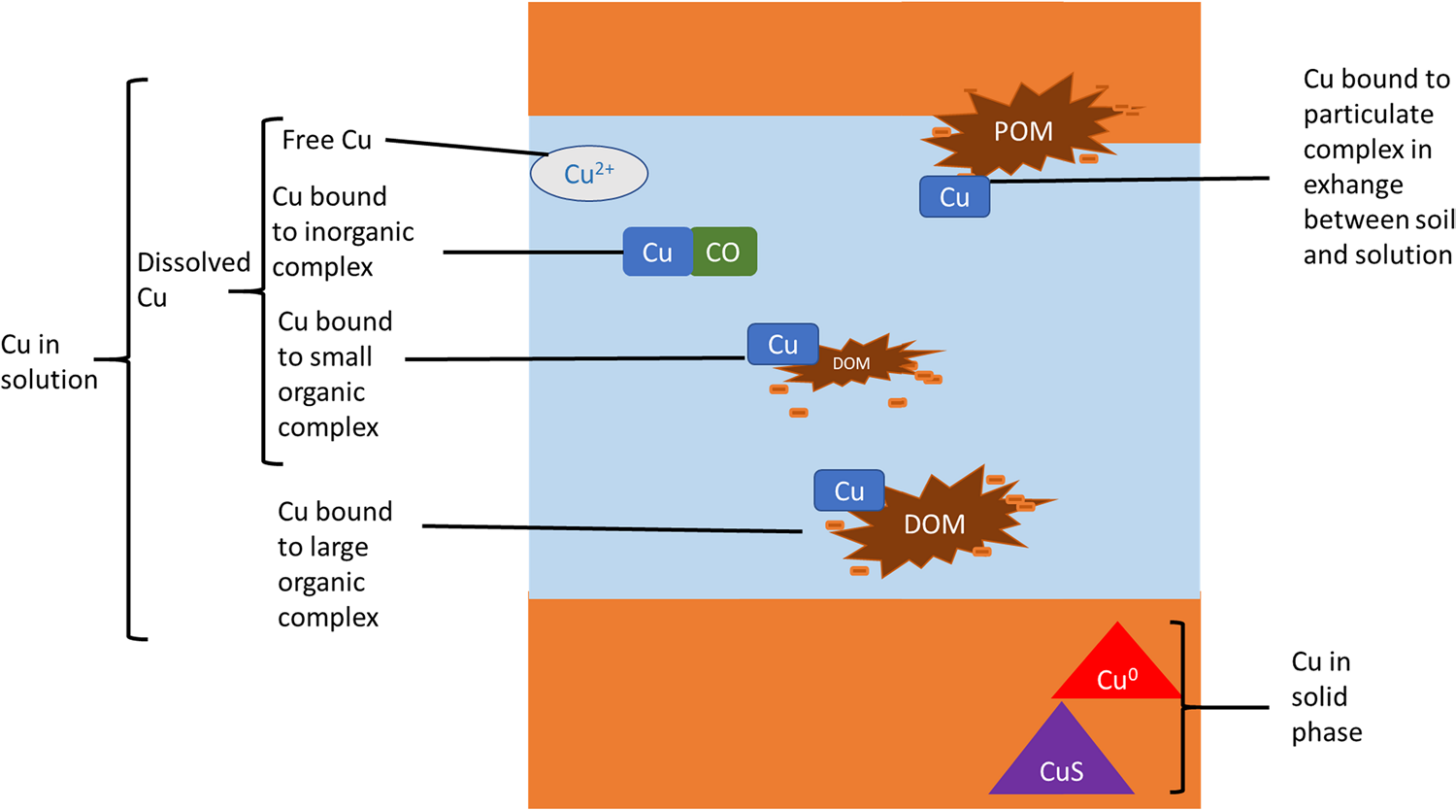
Schematic view of the different forms of Cu in soil systems, POM being particulate organic matter in the solid phase to which Cu can be sorbed, DOM being dissolved organic matter in the soil solution, which binds Cu

Indeed, total soil Cu content can be schematically divided into a pool of sorbed Cu on the solid phase and a pool of Cu present in the liquid phase, both in equilibrium. Cu in solution can also be divided into a pool of Cu complexed to either organic or mineral species and a pool of Cu in the free Cu^2+^ form (e.g., Cu in solution not bound to organic nor to mineral anions; see Fig. 1). Concerning this later pool, the free ion activity model (FIAM) argues that the free form of a trace metal (M) element as M^n+^ is the most biologically impacting form (Parker et al. 2001). Thus, the small and labile fraction of free Cu can be used to advantage as a proxy of bio-available Cu (Lanno et al. 2004; Thakali et al. 2006). However, the knowledge of the total amount of Cu in solution is also important because it is the most likely total pool of Cu that can easily exchange and be available for organisms or for exportation through runoff. Total content of Cu in solution is therefore assimilated to a pool of environmentally available Cu. But when the total trace element content in soil is the only available data, this value is used by default to express the risks even if overestimated (Ministry of the Environment 2007; Oorts et al. 2006b; Smolders et al. 2009). Several studies have underlined the importance of the knowledge of soil parameters (organic matter content, pH, ionic strength, or dissolved organic carbon) to calculate the Cu speciation, i.e. the repartition of Cu in its different forms (Degryse et al. 2009; Mondaca et al. 2015; Sauvé et al. 2000b). In the literature, three main ways can be identified to calculate total Cu in solution and/or free Cu^2+^ forms from the knowledge of total soil Cu content, two of them being based on empirical statistical relationships and one of them on thermodynamic mechanistic models.

From a mechanistic point of view, thermodynamic models allow the calculation of the speciation of Cu in solution using the total soil Cu content and a detailed composition of the soil solution with organic and inorganic compounds as input data. This is currently made with models such as WHAM or Vminteq (Kinniburgh et al. 1996; Tipping 1998) which iteratively compute total Cu concentration under its different chemical forms providing the knowledge of the equilibrium constants of all the potential species. Many hypotheses have to be made to take into account the polyelectrolytic nature of organic matter, the surface geometry, and the electrostatic interactions for complexation and adsorption processes. If such modellings allow a detailed and precise estimation of the different forms of Cu in solution, their use at large scale is complex and challenged due to the number of input data needed. For that, empirical equations can be advantageously used compared to thermodynamic modelling when all the different data are not available.

Empirical relationships result from statistics regressions based on large field-data sets. One approach is to estimate coefficients of partition between solid and solution phases or between solid and free Cu, the other to estimate directly either Cu in solution or free Cu as a sum of several soil parameters. For our purpose, the use of coefficient of partition is few robust because of the assumption that the different forms of Cu are at the equilibrium. Hence, we rather focused on the direct expression of Cu in solution or of free Cu. Numerous empirical equations have been developed to estimate Cu in solution or free Cu based on local measurements or data collections, and variables are traditionally log transformed with a generic expression as following: (Groenenberg et al. 2010)

1 $${\mathrm{log}}_{10}{\mathrm{Cu}}_{f}{=c}_{0}+{c}_{1}{\mathrm{log}}_{10}\left({\mathrm{Cu}}_{\mathrm{total}}\right) +\sum {c}_{i}{\mathrm{log}}_{10}\left({X}_{i}\right)$$

where *X*_*i*_ are the different soil parameters and *f* is the form of Cu (free or in solution) considered.

Recent soil survey from the Joint Research Center (JRC) was performed, and maps of total metal soil content at the European Union scale were produced. These maps underlined large diffuse soil Cu contamination with some hot spots of high total soil Cu concentrations (Ballabio et al. 2018). Applied to Cu, the application of the previous equation could fill the gap of the absence of knowledge of the large-scale distribution of Cu availability, using Cu in solution as a proxy, and the absence of knowledge of the large-scale distribution of Cu bio-availability, using free Cu^2+^ as a proxy. Indeed, availability or bio-availability are data still not documented despite their importance for risk estimation and land management.

In this context, the aim of this work was threefold: (1) provide a literature review of statistical empirical relationships established for estimation of available Cu (Cu in solution) or bio-available Cu (free Cu); (2) estimate, at the European scale, areas of potential risks e.g. of Cu environmental availability or of Cu biological toxicity; and (3) link, at the European scale, the risk due to the presence of a soil Cu contamination to the risk of Cu availability and of Cu bio-availability. We decided not to explore the use of mechanistic models but rather to focus on empirical equations easier to use at larger spatial scales. Based on the literature review, we choose the more appropriate relationships in the objective of application to a European database of total Cu measurements. We highlighted areas of risks of (i) available Cu (Cu in solution) and (ii) bio-available Cu (free Cu) with the comparison of the two values at each grid point with their respective median. This allowed us to define areas of risks without using debated threshold values (Carlon 2007). Furthermore, the use of relative variations through comparisons with median limited misinterpretation due to intercept effects in the chosen empirical equation and allowed underlining the effects of pedological factors. Finally, we identified areas with conflicting or converging risk assessment of availability or of bio-availability compared to the total Cu risk assessment.

## Material and methods

### Equation’s review

In order to provide estimates of Cu forms relevant for risks assessment at the European scale, we collected empirical equations from the literature estimating Cu in solution or free Cu using a two steps approach. We first ran (at the date December 2020) bibliographic research on WOS looking for Cu AND (availab*) AND soil AND TOPIC function. We then completed this research using the references of the collected articles. This allowed gathering the relationships to estimate Cu in solution and free Cu on the basis of soil pedogeochemical characteristics. We only selected relationships using pedogeochemical characteristics commonly measured such as soil organic matter (OM) or soil organic C, dissolved organic carbon (DOC), cationic exchange capacity (CEC), clay percentage, and pH.

Statistical empirical equations mostly provided estimations of Cu content in solution and free Cu concentrations based on total Cu content and other soil parameters. Some empirical equations estimated the so-called “dissolved” trace metal i.e. trace metal in solution after filtration at 0.45 µm. But contrarily to other heavy metals, few Cu is associated to large colloids removed with filtration (Jensen et al. 1999). Since our study focused on the application of transfer functions to estimate (bio-)available risks, we focused on the application of equations for Cu in solution and for free Cu including both the calculation of Cu in solution or dissolved Cu that we considered equally.

To provide a generic guide to select empirical equations while reviewing, we listed the transfer functions together with (1) measurement protocols to acquire Cu data, (2) the number of data used to establish the statistical relationship, (3) their associated R2, (4) the range of soil properties used to define the relationships, (5) the number of times they have been cited, and (6) the number of citations per year. Indeed, among papers, the protocols to acquire Cu data were not uniforms. Measurements of total soil Cu content were made using different methods, (i) after a total HF soil mineralization thus including Cu pedological background or (ii) after a “pseudo-total” soil digestion, thanks to aqua regia or (iii) after a 0.43-M HNO_3_ extraction. It is recognized that the two last extractions approximate total Cu soil content (ISO 2006; Sastre et al. 2002; USEPA 2007). The dilute acid extraction has also been established as an ISO 17586:2016 norm to analyze potential environmental available trace elements. Similarly, to determine Cu in solution, we found in the collected papers several methods to extract soil solution while various types of extraction are known to give different kinds of soil solution (Weihermüller et al. 2007). Finally, because the experimental free Cu measurement requires specific equipment (a selective Cu electrode or a device with Donnan membrane (Minnich and McBride 1987; Pampura et al. 2006)), several studies used theoretical results from speciation modelling software rather than direct experimental measurements.

### Estimation of (bio)-available Cu maps

In this study, we used the European soil Cu survey from the LUCAS database provided by the JRC from which total Cu is based on the aqua regia protocol (Ballabio et al. 2018; Tóth et al. 2016). Hence, we selected from our provided generic guide the transfer functions issued from studies using aqua regia protocols to measure total Cu.

With these collected empirical equations, the estimations of Cu content in solution and of free Cu content in solution allowed building respectively maps of so-called available and bio-available Cu based on pedological mapping provided by the JRC at a 0.5-km scale. The total Cu map was downloaded from https://esdac.jrc.ec.europa.eu/content/copper-distribution-topsoils (Ballabio et al. 2018), pH was downloaded from https://esdac.jrc.ec.europa.eu/content/chemical-properties-european-scale-based-lucas-topsoil-data (Ballabio et al. 2019), clay values were obtained from https://esdac.jrc.ec.europa.eu/content/topsoil-physical-properties-europe-based-lucas-topsoil-data (Ballabio et al. 2016), and total organic carbon (Corga) data were obtained from https://esdac.jrc.ec.europa.eu/content/topsoil-soil-organic-carbon-lucas-eu25 (de Brogniez et al. 2015) and are represented in suppl. Figure 1 and 2, respectively, for soil Corga and pH. Soil OM values were converted to soil Corga content using Corga = OM/2 (Pribyl 2010). For computational time purpose, we used the climate data operator software cdo (Schulzweida 2017) to remap at 0.01° the data originally at the 0.5-km scale.

### Risk assessment

For each proxy associated to Cu (available, bio-available, and total soil content), high-risk areas were identified by computing a risk indicator (RI) in % defined through a comparison with the median value (Eq. 2).

2 $$RI_f = \frac{|{{\mathrm{Cu}}_{f,k}|}-{|\mathrm{Cu}}_{f, \mathrm{Med}}|}{{|\mathrm{Cu}}_{f, \mathrm{Med}}|}\times 100$$

where *f* is the proxy of Cu risk (available, bio-available or total), ${|Cu}_{Med}|$ is the absolute value of its median value, and |Cu_*f,k*_| is the absolute value of the form *f* of Cu for the grid point *k*. *RI*_*f*_ are represented in suppl. Figure 3, 4 and 5 for total, available, and bio-available Cu respectively.

We chose the median rather than the mean value as the reference because of the presence of very few points having high Cu values that pushed up the average (see “Selected regression and computed maps”). Following Reimann et al. (2005), we also identified areas with concentrations of total Cu, available Cu, and bio-available Cu smaller or higher than the median ± 2 times the median average deviations.

The relevance of total Cu to assess soil risk was checked by the comparison of *RI*_total_ with *RI*_*f*_ (with *f* = Cu available or bio-available). Three main classes were defined for total Cu with a risk index higher, lower, or similar to the risk index of the (bio-)available forms, together with 5 subdivisions cases as following:

1. RItotal ≫ *RI*_*f*_
  (A) *RI*_total_ > 0 and *RI*_*f*_ < 0
  (B) *RI*_*f*_ < *RI*_total_ and *RI*_*t*otal_ − *RI*_*f*_ > median (*RI*_total_ − *RI*_*f*_) + 2 × mean average deviation (*RI*_total_ − *RI*_*f*_)
2. RItotal ≪ RI_f_
  (A) *RI*_total_ < 0 and *RI*_*f*_ > 0
  (B) *RI*_*f*_ > *RI*_total_ and *RI*_*f*_ − *RI*_*t*_ > median (*RI*_total_ − *RI*_*f*_) + 2 × mean average deviation (*RI*_total_ − *RI*_*f*_)
3. *RI*_total_ ~ *RI*_*f*_ defined as |*RI*_total_ − *RI*_*f*_|< median (*RI*_total_ − *RI*_*f*_) + 2 × mean average deviation (*RI*_total_ − *RI*_*f*_)

These classes and their subdivisions were defined to highlight the areas where risk assessment based on total Cu differs from those based on available Cu or on bio-available Cu.

The first class (*RI*_total_ ≫ RI_f_) refers to cases where the calculations from the grid points indicated that soil may be considered at risk when considering total Cu measurements but not considering (bio)-available Cu (depending on the *f* Cu form). For 1 (A), the soil may be considered at risk when considering total but not (bio)-available Cu. For 1 (B), the soil is considered at risk for the two indicators, but the risk may be largely underestimated considering (bio)-available Cu in comparison to total Cu. The second class (*RI*_total_ ≪ *RI*_*f*_) refers to cases where soil may be considered without risk when considering total Cu but at risk considering (bio)-available Cu. For 2 (A), the soil may be considered without risk when considering total Cu content but at risk when considering (bio)-available Cu. For 2 (B), the soil is considered at risk for the two indicators but the risk may be largely underestimated considering total Cu in comparison to (bio)-available Cu. The third situation (*RI*_total_ ~ *RI*_*f*_) refers to cases where the differences between total and (bio)-available Cu content are rather small.

Maps and statistical analysis were calculated using R version 3.5 (R Core Team 2018).

## Results

### Literature review of empirical equations

We collected 29 relationships aiming at estimating (bio-)available Cu using total Cu content and soil parameters from 16 references compiled in Table 1. From those 16 references, 1 was produced as part of a report for the environmental research institute of Wageningen (Alterra) (Römkens et al. 2004) on a Dutch soil survey with rather low Cu concentrations close to the local diffuse agricultural contamination in Cu (maximal values around 321 mg. kg^−1^ of Cu while other equations are built on contamination up to a few thousand mg. kg^−1^ of Cu) and with a significant number of data (416). However, the measurements of available Cu were made with a DTPA extraction rather than with dilute salts and the estimation of the total soil Cu content was based on a 0.43-M HNO_3_ extraction. Although interesting, the data from Romkens et al. were not further investigated in this paper.

**Table 1:** Sources of transfer functions, number of citations on 29.07.2021, measured variables, extraction procedure, and no. of equation (for correspondence with Tables 2 and 3) collected from the selected papers. Cu-ISE = copper ion-selective electrode, DPASV = differential pulse anodic stripping voltammetry

DOI	Authors	Year of publication	Times cited	Yearly citation rate	“Total” Cu measurement	Solution extraction	Free Cu estimation	Response variable	No. of equation in the next tables
10.18174/njas.v28i3.17030	Lexmond et al	1980	114	2.8	HNO_3_, HClO_4_, and H_2_SO_4_ in a ratio of 40:4:1	0.01 M CaCl_2_	Resine extraction	pCu	11
10.1111/j.1365–2389.1997.tb00554.x	McBride et al	1997	882	36.8	Nitric acid microwave digestion and H_2_SO_4_^−^HNO_3_ (1:1 by volume), completing digestion with a few drops of HCIO	Water extract and 0.01 M CaCl_2_	Cu-ISE	pCu; Cu solution	3a–e
10.1023/A:1,018,312,109,677	Sauvé et al	1997	383	16	HNO_3_ microwave	0.01 M CaCl_2_	Cu-ISE	pCu	5a, 5b
10.1021/es9907764	Sauvé et al	2000	1279	60.9	Review of “total”	Water displacement, lysimeter, and water or neutral salt extractions		Cu solution	6
10.1021/es0000910	Vulkan et al	2000	180	8.6	Aqua regia	Soil pore water	Cu-ISE	pCu	13
10.1016/S0269-7491(03)00,058–7	Tipping et al	2003	405	22.5	Nitric and perchloric acids, followed by leaching of the residues with 5 mol l1 HCl, and analysis by ICP–AES	2% HNO_3_	WHAM	pCu	14a–b
10.1021/es030155h	Lofts et al	2004	219	12.8	EDTA	0.02 M CaCl_2_, + data from Tipping 2003, Sauvé 1997	CU-ISE and WHAM	pCu	15
Alterra Report 305, May 2004 http://edepot.wur.nl/16988	Römkens et al	2004	66	3.8	0.43 HNO_3_	0.05 mol L Ca-EDTA	CHARON model	Cu solution; pCu	
10.1016/S1001-0742(06)60,016–8	Luo et al	2006	18	1.2	HF, HClO_4_ and HNO_3_ with a ratio of 3:1:1	0.01 KCl	Electrode (DPASV)	Cu solution; pCu	7a–b
10.1016/j.jhazmat.2005.09.033	Luo et al	2006	84	5.6	HF, HClO_4_ and HNO_3_ with a ratio of 3:1:1	0.01 KCl	Electrode (DPASV)	Cu solution; pCu	8
10.1097/SS.0b013e3181bf2f52	Unanumo et al	2009	12	0.8	Aqua regia	0.01 M CaCl_2_	WHAM	pCu	12
10.1111/j.1365–2389.2009.01201.x	Groenenberg et al	2010	102	9.3	0.43 HNO_3_	0.01 ou 0.02 CaCl_2_	WHAM-and Cu-ISE for partial data	pCu	16
10.1080/09064710.2013.785586	Ivezic et al	2012	6	0.7	1:15 HNO_3_	Water 1:10	WHAM	Cu solution	4a
10.1002/jpln.201400349	Mondaca et al	2015	15	2.5	Were digested in boiling nitric acid followed by perchloric acid addition	0.1 MKNO_3_	Cu-ISE	Cu solution; pCu	9a–c
10.1080/09542299.2017.1404437	Li et al	2017	3	1	Aqua regia	Filtered pore water	Cu-ISE	Cu solution; pCu	10a–c

The oldest equation specifically applied to Cu was provided by Lexmond (1980) (see Eq. 11 in Table 1) to estimate bio-available Cu (expressed as − log(Cu) = pCu), and the last equation we found was designed by Li et al. (2017a, b) (see Eq. 10 in Table 1) to estimate bio-available Cu. Among these 29 relationships, we found 13 equations aiming at estimating specifically the available Cu (Cu in solution) and 16 estimating the bio-available Cu (free Cu). Assuming that yearly rate of citations is a proxy for a scientific consensus and/or the easy-to-use, we found that many studies used the Sauvé et al. (2000a) approach with 61 citations/year (see Eq. 6 in Tables 1, 2, and 3), or the McBride et al. (1997) approach with 37 citations/year (see Eq. 3 in Tables 1, 2, and 3) followed by those of the Tipping et al. (2003) approach with 23 citations/year (see Eq. 14 in Tables 1, 2, and 3). Total soil Cu was the most frequent predictor to calculate available Cu with 11/13 equations using total Cu, while pH was the most frequent predictor to calculate bio-available Cu with 16/16 equations based on pH.

**Table 2:** Transfer functions for Cu available reviewed from literature under the form log_10_Cu_solution_ = a log_10_Cutot + b log_10_OM + c log_10_clay + d log_10_pH + e. Cu is expressed in mg. kg soil^−1^ of Cu, OM is expressed in g. kg soil^−1^ of OM or in % of OM (specified in the row), and DOC is expressed in mg. L^−1^ of C and clay in %. When parameters' incertitudes were provided, they have been reported in the table

Source	No	R.V	*e*		Log (Cu tot)	pH	Log (OM)	Log (DOC)	Log (clay)	R2	Number of data	Range Cu tot (mg. kg^−1^ of Cu)	Range OM (g. kg^−1^ of OM)	Range pH
McBride et al. (1997)	3a	Log (Cu_solution_) (µg. L^−1^)	0.699		0.86	− 0.11				0.87	67	14–2600		3.3–6.6
	3b	Log (Cu_solution_) (µg. L^−1^	1.42		0.94	− 0.1	− 0.68 (g. kg^−1^)			0.85	70	14–2600		3.3–6.6
	3c	Log (Cu_solution_) (µg. L^−1^)	0.05		0.76					0.86	31	7–1010	2.4–27.4	4.2–7.8
Ivezić et al. (2012)	4a	Log (Cu_solution_)	− 0.24		0.80	− 0.02	− 0.53 (%)	0.54		0.42	74	5.7–141	1.8–20.4	4.3–8.1
	4b	Log (Cu_solution_) (µg. L^−1^)	− 0.45		0.77		− 0.62 (%)	0.65		0.42	74	5.7–141	1.8–20.4	4.3–8.1
Sauvé et al. (1997)	5a	Log (Cu _solution_) (µg. L^−1^)		13.2 (± 7.9)	0.32 (± 0.01)					0.89	66	14–3083	4.1–554.6	3.3–7.6
Sauvé et al. (2000a, b)	6a	Log (Cu _solution_)		1.37 (± 0.14)	0.931 (± 0.05)	− 0.21 (± 0.02)	− 0.21^a^ (± 0.02)			0.611	353			2–9
Luo et al. (2006b)	7a	Log (Cu _solution_) (µg. L^−1^)		1.21 (± 0.45)	0.32 (± 0.16)		1.08 (± 0.33) (%)			0.32	39	280–1752		5.3–7.61
	7b	Log (Cu _solution_) (µg. L^−1^)		2.08 (± 0.11)			1.33 (± 0.32) (%)			0.38	39	280–1752		5.3–7.61
Luo et al. (2006a)	8a	Log (Cu _solution_) (µg. L^−1^)		2.20 (± 0.11)			0.88 (± 0.28) (%)			0.205	40	280–1930	26–62	5.5–7.8
Mondaca et al. (2015)	9a	Log (Cu _solution_) (µg. L^−1^)		0.69	0.5		0.73			0.36	86	56–4441	12.0–62	6.2–7.8
	9b	Log (Cu _solution_) (µg. L^−1^)		− 1.01	0.75		0.95		1.23	0.70	86	56–4441	12.0–62	6.2–7.8
Li et al. (2017a, b)	10a	Log (Cu _solution_) (µmol. L^−1^)		− 2.976	0.515				1.23	0.63	34			

^a ^Data expressed in percentage of C ^b^ Data expressed in mg.kg^−1^ soil^−1^ of Cu

**Table 3:** Transfer functions for bio-available Cu reviewed from literature for estimation of pCu (units in brackets). pCu = a log_10_Cutot + b log_10_OM + c log_10_clay + d log_10_pH + e. R.V is for response variable and e. for intercept. Total Cu is expressed in mg. kg^−1^ of Cu, OM in g. kg^−1^ of OM or percentage (precision in the row), and clay in percentage

Source	Eq	R.V	*e*	Log (Cu tot)	pH	Log (OM)	Log (clay)	R2	Number of data	Range CuTot (mg. kg^−1^ of Cu)	Range OM (g. kg^−1^ of OM)	Range pH
Lexmond (1980)	11	pCu (mol. L^−1^)	5.08	− 2.38	1.07^a^			0.989	16	10–400	16.8	3.9–6.2^b^
(McBride et al. (1997)	3d	pCu (µg. L^−1^)	1.28	− 1.95	1.37	1.95 (g .kg^−1^)		0.897	70	17–2600		3.3–6.6
McBride et al. (1997)	3e	pCu (µg. L^−1^)	1.8	− 1.1	1.6	1.8 (g. kg^−1^)		0.91	10	6–1440	15–395	4.5–7.2
Sauvé et al. (1997)	5b	pCu (µg. L^−1^)	3.42 (± 0.5)	− 1.7 (± 0.12)	1.4 (± 0.08)			0.848	66	14–3083	4.1–554.6	3.3–7.6
Unamuno et al. (2009)	12a	Log (Cu^2+^) (mg. kg^−1^)	− 2.1		0.085		0.005	29	18–10,389			
Unamuno et al. (2009)	12b	Log (Cu^2+^) (mg. kg^−1^)	− 2.079	0.593	− 0.053		0.73					
Unamuno et al. (2009)	12c	Log (Cu ^2+^) (mg.kg^−1^)	− 2.259	0.594	− 0.058	0.09 (g kg^−1^)	0.732	29	18–10,389			
Vulkan et al. (2000)	13a	pCu (µg. L^−1^)	− 0.53	− 1.47	1.79			0.89	22	19.4–8645	98–698	5.5–8
Tipping et al. (2003)	14a	pCu (µg .kg^−1^)	− 1.34	− 0.54 (µmol. g^−1^)	1.15	0.40 (%)		0.94	98		100–1000	
Tipping et al. (2003)	14b	pCu (µg. kg^−1^)	− 5.35	− 1.09 (µmol. g^−1^)	1.17	0.52 (%)		0.87	165			
Lofts et al. (2004)	15	pCu (nM)	− 4.99	− 0.93	1.26	0.63(%)		0.9	151	0.96–637	0.41–97.8	3.35–8.27
Luo et al. (2006b)	7b	pCu (µg. L^−1^)	− 2.24 (± 0.96)		1.47 (± 0.14)			0.76	39	280–1752		5.3–7.61
Groenenberg et al. (2010)	16	pCu (mol. L^−1^)	0.48	0.81	− 1	− 0.89 (%)	0.83	216	0.6–326	2–97.8		3.3–8.3
Mondaca et al. (2015)	9c	pCu (µg. L^−1^)	5.54	− 0.74	0.67	0.75(%)		0.58	86	56–4441	12.0–62	
Li et al. (2017a, b)	10b	pCu (mol. L^−1^)	− 4.303	− 1.639	1.171			0.65	34			
Li et al. (2017a, b)	10c	pCu (mol. L^−1^)	− 0.783	− 1.6 log(Cu solution, µg. L^−1^)	1.3 log(Cu solution, µg L-1)			0.65	34			

^1^pH in the resin extraction

^2^pH in soil solution determined by CaCl_2_

#### Selection of the empirical equation to build the available Cu map

Table 2 provides the collected 15 equations of the literature estimating the amount of Cu in solution used here as a proxy for available Cu, and taken into account soil properties. The corresponding soil solution extraction and total Cu mineralization methods are reported in Table 1. Total soil Cu content is the most frequent explaining variable, found as a reliable predictor for all except one relationship. All the relationships showed that available Cu decreases when soil pH increases, so that there are more available Cu under acid soil conditions. DOC’s partial slope is mostly found as non-significant or positive, indicating that dissolved organic carbon can bind Cu in solution through organic complexes On the other hand, the equation performed for Cu by Sauvé et al. (2000a) was fitted on more than 350 data collected in the literature, and seem to be the most robust empirical equations in estimation of dissolved Cu. It is also the most cited equation preferentially used to convert a large range of soil Cu total content into dissolved Cu values. The willing to include as much data point originating from various databases is however coupled with the lack of information about the measurement techniques involved. In fact, “total” Cu is mentioned without specifying the soil digestion method. In complement, we also noted the empirical equation of Mondaca et al. (2015) (Table 2, Eq. 9) which was fitted with data from Chili and can thus be more appropriate for semi-arid region and their typical pedological characteristics and climate compare to Europe (Garcia et al. 2017; Steven 2017). Finally, among the collected equations, those from McBride et al. (1997) (Table 2, Eq. 3 a-c) are among the most commonly used with more than 36 citations/year. The authors provided two main regressions with exclusion or inclusion of data points (Table 2, Eq. 3a.) with highest (> 100 g. kg^−1^) OM content (Table 2, Eq. 3b.). Equations 3 a, b were built on the basis of a 70-point dataset including a long-term contamination due to sludge inputs or industrial activities deposition. For Eq. 3a (Table 2), a maximal total soil Cu concentration around 3000 mg. kg^−1^ of Cu was achieved, whereas Eq. 3c was based on a dataset with Cu contamination from 7 to 1000 mg. kg^−1^ of Cu. Total soil Cu concentrations were measured with acid micro-wave digestion providing values close to aqua regia extraction, whereas available Cu values came from 0.01 M CaCl_2_ extractions (Eq. 3 a, b) or water extractions (3c). Moreover, all the variables of the equations are available in the LUCAS database we intend to use. We therefore selected Eq. 3b that fitted more data points to calculate the available Cu at the European scale.

#### Selection of the empirical equation to build the bio-available Cu map

Considering that bio-available Cu can be approximated by the content of free Cu in solution, we gathered the equations predicting pCu (= − log_10_[Cu^2+^]) from literature which are reported in Table 3. We took into account an important parameter when comparing the equations, specified in Table 1: the fact that bio-available Cu is experimentally measured or theoretically predicted by speciation software. Ten studies were based on measurements and six on modelling. In all the resulting empirical equations, pCu is negatively correlated to total soil Cu content and positively correlated to pH and OM. This means that there are more bio-available Cu when the total soil Cu content is high and when the soil organic content is low. Interestingly, in almost all the empirical equations with pCu, the parameters associated to pH and total Cu coefficients are roughly of equal importance. On the contrary, the parameters associated to total Cu are from 4 to 40 times more important than that of pH in relationships to calculate available Cu.

McBride et al. (1997) and Tipping et al. (2003) reported the most commonly used equations to determine free Cu. Their regressions were based respectively on 70 (Table 3, Eq. 3d, 3e) and 165 samples (Table 3, Eq. 14a, 14b.) from long-term contaminated soils with a large range of contamination going up to 3000 mg. kg soil^−1^ of Cu. It is important to note that the two studies used the same data set than that of Sauvé et al. (1997) for the Eq. 5b (Table 3). McBride et al (1997) built their regression on the 70 data of Sauvé et al. (1997) with inclusion of pH, total Cu and OM. This last parameter was excluded by Sauvé et al. (1997) in the equation they proposed (Eq. 5b) because it was shown to be strongly related to soil Cu content. Tipping et al. (2003) proposed an equation using an extension of the Sauvé’s dataset adding 98 points from moorland soils (Table 3, Eq. 14b). They also provided an empirical equation restricted on the moorland soils (Table 3, Eq. 14a) which can be particularly useful for soils with high OM content; this parameter has been found to significantly impact Cu availability and equation’s parameter values. Finally, we chose to use Eq. 14b from Tipping et al. (2003) since it was based on the largest dataset and the pCu data were measured and not estimated using a mechanistic model.

### Application to Europe mapping

#### Selected regression and computed maps

**Fig. 2:**
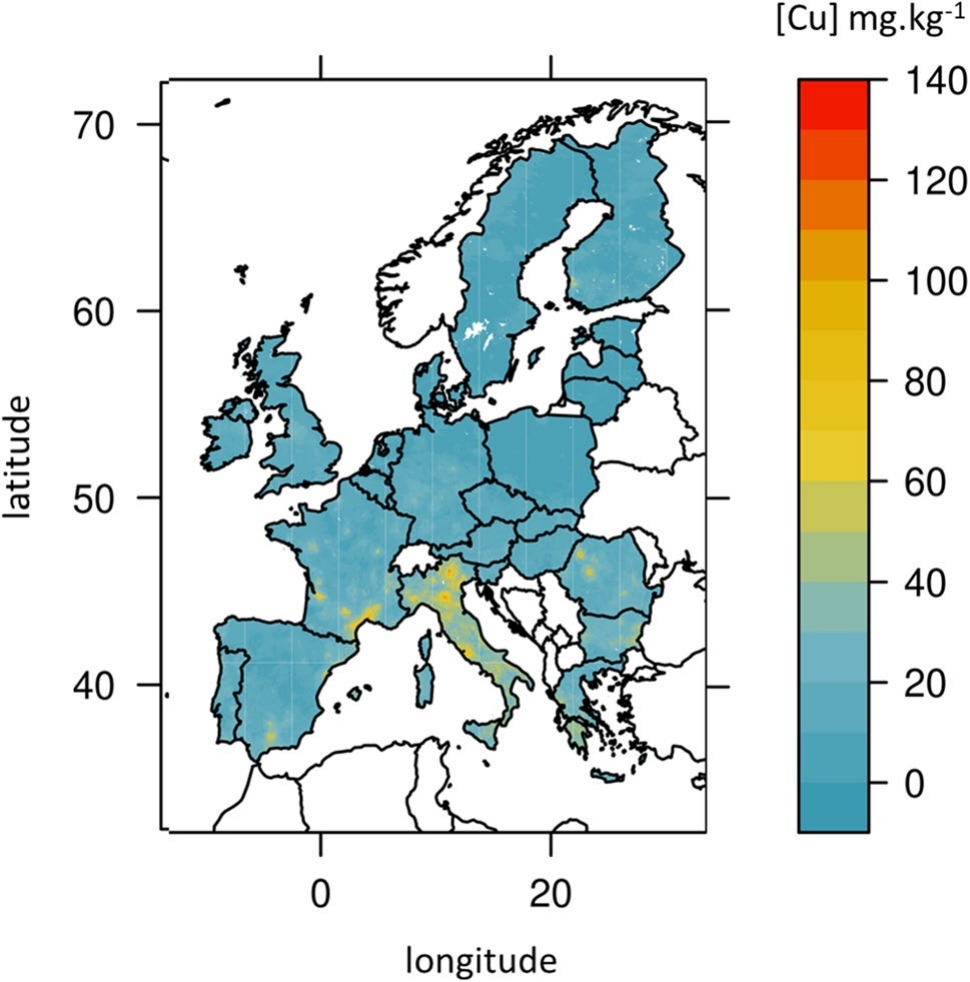
European map of total Cu in soils (mg.kg soil^−1^ of Cu) after conversion at 0.01°, using the data from the JRC, extracted from https://esdac.jrc.ec.europa.eu/content/copper-distribution-topsoils (Ballabio et al. 2018)

The total Cu concentration in the LUCAS database provided by the JRC varied from 1 to 130 mg. kg soil^−1^ of Cu with most (75%) of the values below 20 mg. kg soil^−1^ of Cu and 99.9% below 52 mg. kg^−1^ of Cu (Table 4; Fig. 2). With the definition of geochemical baselines as values in the range of median ± two times the average deviation (Reimann et al. 2005), we considered that 1.5% of the soils are over-concentrated with a total soil Cu content > 28 mg. kg^−1^ of Cu, and that none are depleted. The range of risk index for total Cu was *RI*_total_ [− 94 to 883%]. Roughly, 25% of the grid points had *RI*_total_ > 50% meaning a total soil Cu content higher than two times the median European value. Furthermore, less than 10% of the grid points had *RI*_total_ > 100% and around 10% of the grid points had *RI*_total_ < − 50% (suppl table 2).

**Table 4:** Deciles of concentration for total soil Cu contents (mg. kg^−1^ of Cu), Cu in solution (µg. L^−1^ of Cu), and free Cu (mg. kg^−1^ of Cu), also expressed as pCu (− log(freeCu))

decile	0.00	0.01	0.05	0.10	0.15	0.20	0.25	0.40	0.50	0.60	0.70	0.75	0.8	0.90	0.95	0.99	1.00
total Cu	0.81	3.44	5.03	6.25	7.23	8.08	8.89	11.36	13.21	15.35	17.94	19.56	21.65	28.38	36.8	28.38	129.95
Cu solution	0.20	0.68	1.01	1.26	1.45	1.63	1.83	2.63	3.36	4.17	5.08	5.64	6.33	8.49	10.71	15.60	45.05
pCu	− 2.01	− 0.78	− 0.45	− 0.28	− 0.15	− 0.04	0.07	0.49	0.78	1.07	1.38	1.56	1.77	2.25	2.58	3.14	4.02
free Cu	9.48 E-05	0.001	0.003	0.006	0.010	0.017	0.028	0.085	0.165	0.324	0.636	0.845	1.095	1.892	2.823	5.965	101.771

**Fig. 3:**
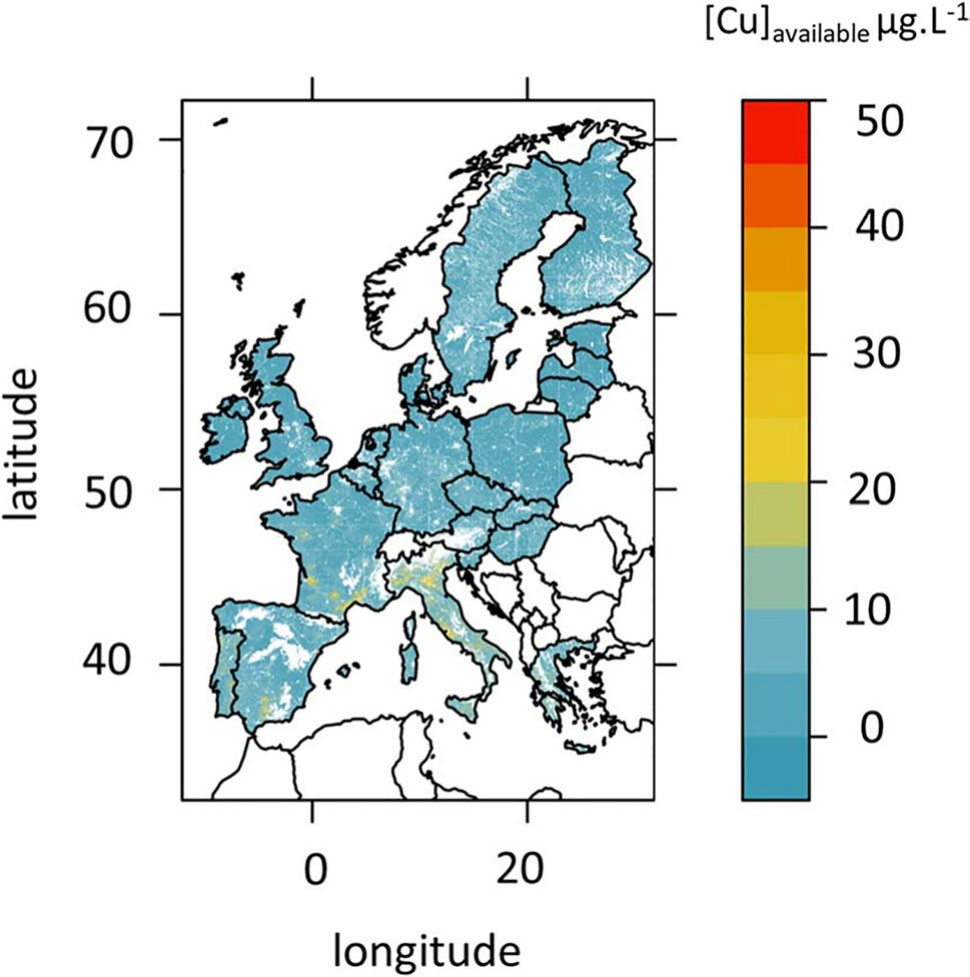
European map of available Cu (taken Cu contents in soil solution as a proxy in µg. L^−1^ of Cu) at 0.01° estimated using the empirical equation of McBride et al (1997) (Eq. 3b, Table 2) and the map of total Cu (Fig. 1) with pH and Corga provided by the JRC. Vertical color scale is for available Cu concentrations (µg. L^−1^ of Cu)

**Fig. 4:**
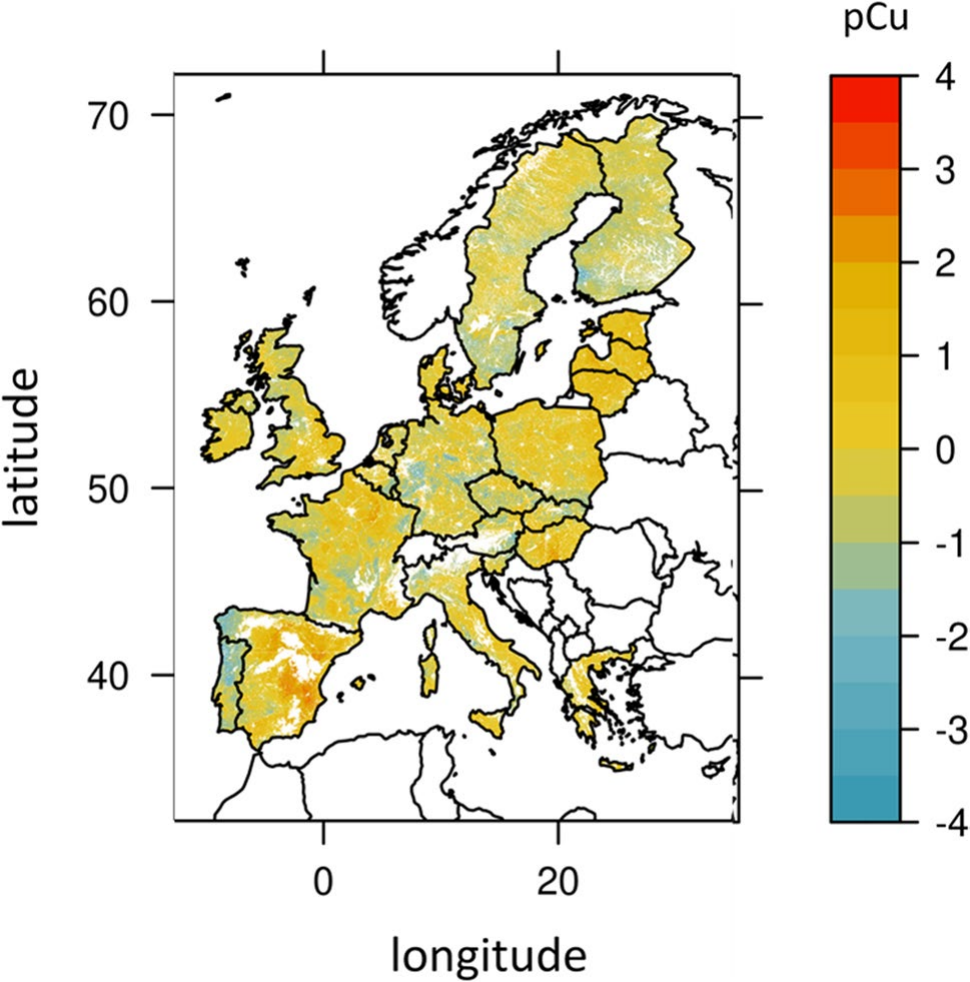
European map of bio-available Cu (taken free Cu contents in soil solution as a proxy expressed in term of pCu) at 0.01° based on Tipping et al. (2003) empirical equation (Eq. 14b, Table 3) and the map of total Cu, and with pH and Corga provided by the JRC. Vertical scale is for bio-available Cu concentration expressed in pCu = − log[Cu.^2+^]

Based on the choice of transfer functions and the data provided by the JRC, we calculated and edited two different maps at the European union scale: one of the available Cu based on the McBride et al (1997) estimation (eq. n° 3b) to derive available Cu in solution (Fig. 3) and one of the bio-available Cu based on the Tipping et al. (2003) regression to derive pCu (eq n°14b) (Fig. 4).

Due to the lack of Corga measurements in mountain soils, there is part of the European territory without estimation of (bio-)available Cu. Estimation of available Cu varied from 0.2 to 45 µg. L^−1^ of Cu with 75% of the values below 5.6 µg. L^−1^ of Cu and 99% below 15 µg. L^−1^ of Cu (Table 4). With the definition of geochemical baselines as values in the range of median ± two times the average deviation (Reimann et al. 2005), 10.% of the soils are considerate as over-concentrated with available Cu > 8.5 µg. L^−1^ of Cu and none are considered as depleted.

Bio-available Cu varies from 1.79 × 10^−9^ to 0.002 mg. kg^−1^ of Cu with 75% of the values below 2.80 10^−5^ mg. kg^−1^ of Cu and 99% below 1.80 10^−4^ mg. kg^−1^ of Cu (Table 4). 1.87% of the grid points have bio-available Cu defined as below the geochemical baseline and 0.01% above.

An area of high concentration for one form of Cu is not systematically highly concentrated if we considered another Cu form. For instance, the region with higher bio-available Cu (pCu < − 0.4, 95% decile, in North West Spain or Austria) have total Cu ranging from 2.2 (< 1% decile) to 90 mg. kg^−1^ of Cu soil (99% decile) and available Cu from 0.6 (1% decile) to 35.6 µg. L^−1^ of Cu (> 99% decile) (suppl. table 1). In this example, high bio-available Cu is linked to low total Cu or low available Cu, highlighting that the three proxies do not necessarily follow the same pattern.

#### Spatialization of available Cu risks and comparison of risk index with the total Cu map

The range of variations compared to the median values is similar between total Cu and available Cu (suppl. Table 2 and 3). For available Cu, the range of *RI*_available Cu_ is [− 94 to 1241%]. Roughly, 30% of the grid points had *RI*_available Cu_ > 50%, 10% had *RI*_available Cu_ > 150%, and 20% had *RI*_available Cu_ < − 50% (suppl table 3). Total Cu and available Cu show similar pattern of highest/lowest concentrations (suppl. Figure 4 and 5). Highest concentrations are mostly found in Eastern Europe, South of Spain, Portugal, parts of West France, and of England, but there are also some small areas in North West of Norway and South of Sweden (Figs. 2 and 3).

**Fig. 5:**
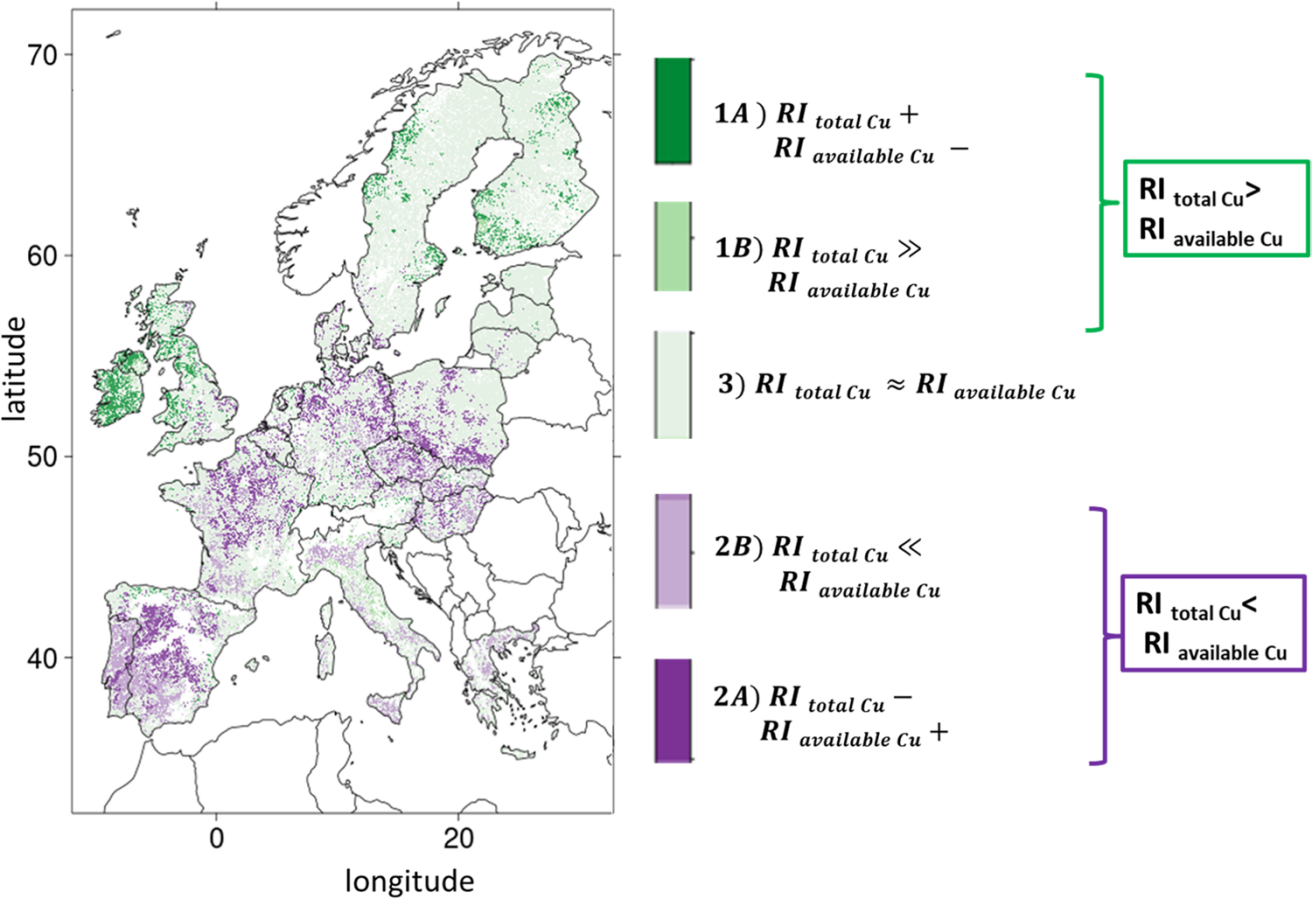
Map of risk assessment showing the comparison between total and available Cu at 0.01° based on the *RI*_total Cu_ and *RI*_available Cu_ following the 5 cases defined in “Risk assessment”, from purple meaning an underestimation of risk based on total rather to available Cu to green meaning an overestimation of risk based on total rather than to available Cu

The comparison between *RI*_available Cu_ and *RI*_total_ is shown in Fig. 5. This map highlights important differences in risk consideration. Only 6.0% of the grid points fitted with situation 1 as described in the material and method section, indicating that the risk assessment based on total Cu may be overestimated compared to the risk assessment based on available Cu. Indeed, we have *RI*_total_ ≫ *RI*_available Cu_ for 5.3% of the grid points (case 1A) and opposite signs with positive *RI*_total Cu_ for 0.7% of the grid points (case 1B). This was mainly assessed for Ireland, North West of Norway, and South of Finland and for isolated points in Germany. More grid points (19.6%) fitted the situation 2, indicating that risk assessment based on total Cu is underestimated compared to risk assessment based on available Cu. Indeed, we have *RI*_total_ ≪ *RI*_available Cu_ for 10.7% of the grid points (case 2A) and *RI*_total Cu_ < 0, but *RI*_available Cu_ > 0 for 8.9% of the grid points (case 2B). These situations mostly occurred in central Spain, central France and North East Germany, south Spain, Italy, and central East Europe. Comparable RI for total and for available Cu (see situation 3 in “Risk assessment”) were mostly found in Scandinavia, Brittany (France), Germany, and central Italy. As a consequence, 25.6% of the grid points present discrepancies when risk assessment is based on total Cu or based on available Cu.

#### Spatialization of bio-available Cu risks and comparison of risk index with the total Cu map

The computed map of bio-available Cu expressed as pCu is given in Fig. 4. This map differs from the map of total Cu (Fig. 2) and that of available Cu (Fig. 3) with hotspots in Galicia (North West of Spain), parts of West England, and Roma region but no other areas in Italy. Germany and West ex former Union present high bio-available Cu concentrations despite relatively low total Cu (Figs. 2 and 4). High bio-available Cu contents observed in West Iberian, Germany, and South Scandinavia coincide with areas of low pH and emphasize the strong dependency of bio-available Cu to pH. Indeed, because pH is high in the Adriatic coast of Italy and Lombardy, there are low bio-available Cu content despite relatively high soil total Cu content (Figs. 2 and 4; suppl. Figure 2). The range of variations in bio-available Cu are largest than the variations in total or in available Cu with *RI*_bio-available Cu_ varying from [− 99.9 to 61,679%]. Moreover, numerous grid-points are far from the median. Indeed, *RI*_bio-available Cu_ was < − 58% for 40% of the grid points and *RI*_bio-available Cu_ > 1000% for more than 10% of the grid points. For 1% of the grid points, mostly in West Iberia, *RI*_bio-available Cu_ was > 3520% (suppl table 4). Areas of *RI*_bio-available Cu_ are much narrowed than those of *RI*_available Cu_, so that central and East Spain, centre West of France, and North-East coast of Italy would be under- rather than over-concentrated in bio-available Cu. Besides, large parts of Scandinavia have high levels of bio-available Cu (suppl Fig. 4).

**Fig. 6:**
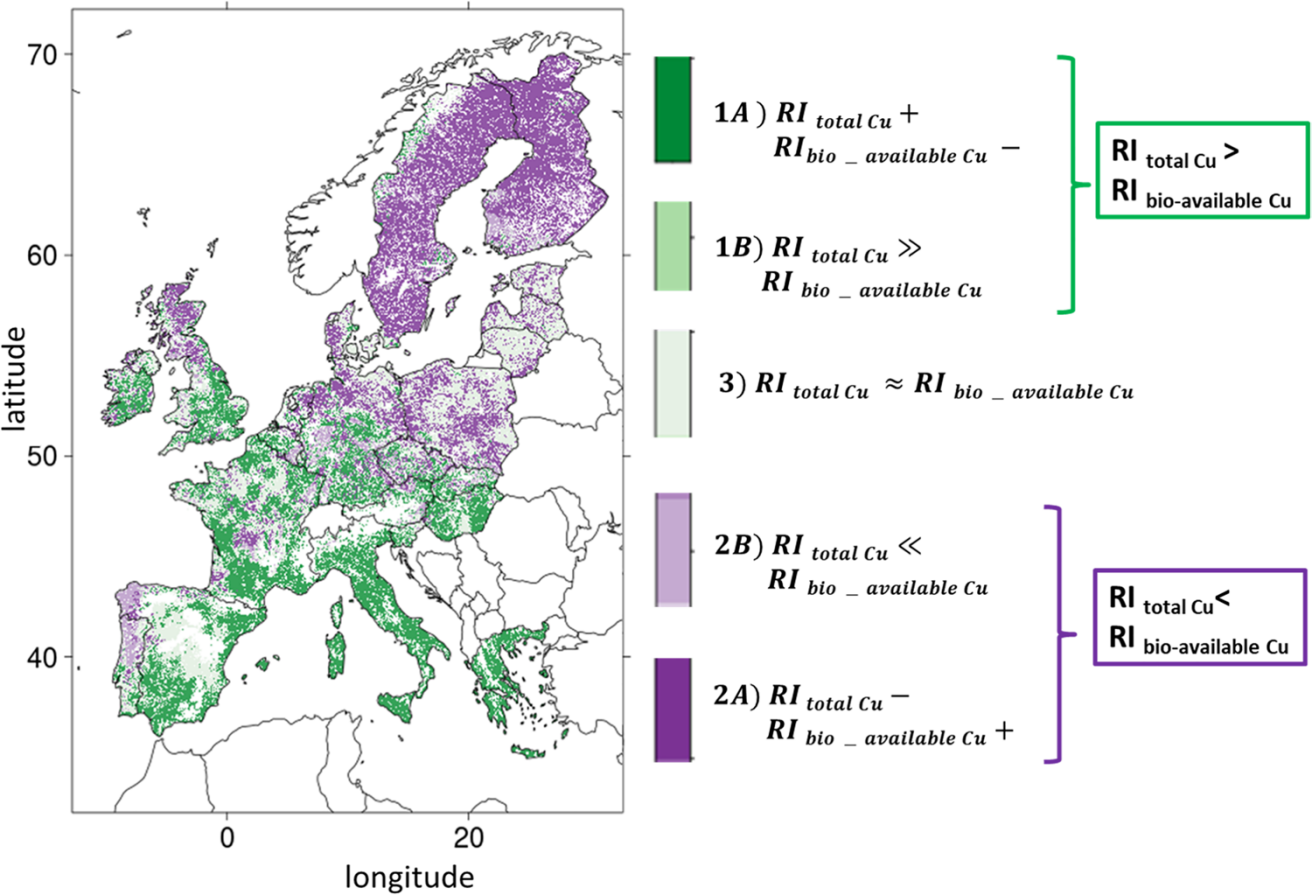
Map of risk assessment showing the comparison between the *RI*_total Cu_ and *RI*_bio-available Cu_ following the 5 cases defined in “Risk assessment”, from purple meaning an underestimation of risk based on total rather to bio-available Cu to green meaning an overestimation of risk based on total rather than to bio-available Cu

The comparison of *RI*_bio-available Cu_ with *RI*_total_ in Fig. 6 shows important differences in risk consideration. 28.9% of the grid points fitted with situation 1, indicating that the risk assessment based on total Cu may be overestimated compared to the risk assessment based on bio-available Cu. Almost all of this 28.9% of the grid-points have *RI*_total Cu_ > 0 but *RI*_bio- available Cu_ < 0 (case 1A in “Risk assessment”). On the contrary, 39.5% of the grid points fitted with case 2 indicating that the risk assessment based on total Cu may be underestimated compared to the risk assessment based on bio-available Cu. In details, 34.3% of the grid points have *RI*_total Cu_ < 0 but *RI*_available Cu_ > 0 (case 2A in “Risk assessment”) and 5.2% RI_total_ ≪ *RI*_available Cu_ (case 2B). These situations mostly occur in Scandinavia, West Iberian Peninsula, and North of Central Europe. Comparable RI for total and for bio-available Cu (situation 3 in “Risk assessment”) were mostly found in central Spain, Poland, Czech Republic, or Slovakia. Therefore, 68.4% of the grid points present discrepancies when risk assessment is based on total Cu instead of bio-available Cu.

## Discussion

### Generic purpose of empirical equations

The present study was mostly focused on the determination of the available Cu pool (assimilated to the Cu content in solution) and of the bio-available Cu pool (assimilated to the free Cu content in solution). We showed that the collected empirical equations were defined on measurements based on different extractions procedures for available Cu. Besides site-specific properties, the differences in experimental procedures can explain the differences in fitted coefficients. There is, however, a good agreement between studies in their selection of variables considering pH, total soil Cu, soil OM, DOC, or clay as key variables to predict (bio-)available Cu. In fact, pH was found to be the most important predictor with 24 empirical equations involving all the forms of Cu (dissolved or free) using pH as a predictor followed by OM and total Cu, whereas CEC or clay were more rarely found as predictors of interest. The importance of pH in cation partitioning is well recognized (Buchter et al. 1989; Flemming and Trevors 1989; Sauvé et al. 2000a) and the effect of pH can be explain with a semi mechanistic approach which assumes that free cation such as Cu^2+^ and H^+^ compete for adsorption on carbonates or OM (Basta et al. 1993; Harter and Naidu 1995; McBride et al. 1997; Bradl 2004). The relative weight of OM, pH or total Cu in empirical equations were however different between equations for available Cu or for bio-available Cu (Tables 2 and 3). Indeed, the log of available Cu increases of roughly 1 unit per unit of increase in total Cu or per 5 units of decrease in pH, while bio-available Cu (pCu) decreases of roughly 1 unit per unit of increase in log of total Cu or per unit of decrease in pH. Thus, due to the low pH in North Spain, Norway, Germany and West England, bio-available Cu is particularly high (i.e. with low pCu values) in these regions despite low total Cu content (around 10 mg. kg soil^−1^ of Cu).

Our results are consistent with the competition between H^+^ and Cu^2+^ for sorption onto soil OM only in the case of available Cu, but for sorption onto both soil OM and DOC in the case of bio-available Cu. However, cation availability cannot be limited to a first order relationship of binding with organic matter because the decrease in available Cu in solution with the increase in pH involves different processes. For instance, above pH 7.7, most of Cu in solution is expected to be found as Cu(OH)_2_ and is about to precipitate (Ma et al. 2006b); on the other hand, studies found that between pH = 4.5 and 7.7, Cu would be retained by ferric hydroxide coated sands (Al-Sewailem et al. 1999). The aim of the empirical equations we collected here was to provide generic equations valid over a wide range of parameters for application at a large scale (Cavallaro and McBride 1978) despite these different processes. Thus, apart complete speciation models which require numerous parameters including the nature of the reactive dissolved organic matter, some equations as those provided by Römkens et al. (2004) complete the classical parameters with Fe or Al oxides content. In their study the improvement is however limited (*r*^2^ from 0.65 to 0.66 when adding Fe and Al oxides) which suggests punctual outliers rather than generic predictors. On the contrary, the good fits of new coefficients with the empirical equations based on a restricted number of predictors (pH, total Cu, soil OM) selected by other studies (Lofts et al. 2004) confirm their genericity consistent with an extrapolation for upscaling to Europe maps. In order to validate our estimations, we looked for studies using local Cu content independent from the data set we used to calibrate the empirical equations. For example, Buccolieri et al. (2010) measured both total Cu and available Cu (as a DTPA-extract) in several sites in South Italy, and found in mean, respectively, 70 mg. kg^−1^ of Cu (from 4.5 to 280 mg. kg^−1^ of Cu) and 5 mg. kg^−1^ of Cu (from 0.38 to 25 mg. kg^−1^ of Cu). From the JRC maps, we can extract for this region a mean of total soil Cu value around 36 mg. kg^−1^ of Cu (values from 12 to 64 mg. kg^−1^ of Cu) and our estimation of available Cu with the empirical relationship was consistent with the experimental value of Buccolieri et al. (2010), with a mean value of 11 mg. kg^−1^ of Cu (values from 3 to 21 mg. kg^−1^ of Cu).

Our results are also in line with the ratio between bio-available Cu (the so-called fraction measured in an EDTA-extract) and total Cu estimated by Tarvainen and Kallio (2002) over Finland who estimated that in mean bio-available Cu account for 7.7% of total Cu. Based on JRC map and our estimation of bio-available Cu, our mean ratio of bio-available over total Cu is 15% with 50% between 0.07 and 13% of total Cu being bio-available.

### What is the usefulness of using Cu in solution or free Cu to characterize risk assessment?

Our results show a large variability in Europe considering all the forms of Cu. For both available and bio-available Cu, we identified patterns of high concentrations at the regional scale. However, in both cases the 1% points that are more concentrated were isolated rather than regionally located, suggesting hot spots. For available Cu, these most concentrated grid points were mostly in North Italy and South East of France but we could not precisely delimitate an area of concern. For bio-available Cu these local hot spots were rather in Austria, North Spain, and South-West Finland. The total Cu survey performed by the JRC (Ballabio et al. 2018) identified that Nomenclature of Territorial Units 2 (NUTS 2, regional scale) was one of the most determinant factor to explain total Cu concentration at the European scale. Thus, wine producing regions have globally high Cu concentration because of the use of “bouillie Bordelaise” for vineyards, but environmental guidelines of each local administration also limit total Cu concentration. In our study, we found that co-factors like soil OM and pH largely affect Cu availability even at a large scale, and that pH was in equal importance than total Cu to explain bio-available Cu variations. In addition, areas like Scandinavia with moderate total Cu but low pH exhibit high bio-available Cu values so that the associated risk is higher with this last proxy.

Besides, with the assumption that Cu in solution could be exported through runoff to downstream ecosystem, the amount of rainfall would be of major importance to consider risk at both local and regional scales (Lefrancq et al. 2014; Xu et al. 2014). Most of the areas with high concentrations of available or bio-available Cu are located around the Mediterranean Sea, where summer are usually dry with intense thunderstorms and cold and wet winters. Climatic scenario forecast a global decrease in precipitation in these regions, but also that rainfall events will be more intense (Christensen and Christensen 2003; Giorgi and Lionello 2008). Thus, if the average export could decrease with a decrease in rainfall, flushes of higher intensities coupled with erosion could arise (Imfeld et al. 2020; van der Knijff et al. 2000). Thus, a new question we may answer is the availability of Cu in the retention ponds where concentrations will increase due to upstream exports. In parallel, we found that bio-available Cu was particularly high in Portugal and Scandinavia where climatic prevision forecasts particularly a high temperature rise, drought for Portugal and higher rainfall patterns for Scandinavia (Christensen and Christensen 2003). These modifications in climate may thus affect plants and soil micro-organisms to the Cu stress and affect their response to soil Cu (J. Li et al. 2017a, b; Tobor-Kapłon et al. 2006). Here, we highlighted that contamination assessment based on total Cu differs from the assessment based on (bio-)available Cu, even at the regional scale. Thus, it might help to take into account the expected climate change to gain in robustness when assessing the evolution of soil contamination.

### Are contaminated soils at equilibrium?

The equations we reviewed here were mostly constructed on data from long-term contaminated soils where Cu species were supposed to be in field at equilibrium. However, in this study, we used total Cu data acquired during field campaigns without precision on the temporality of the Cu inputs nor on the delay after Cu applications. However, several studies show that extractability of Cu decreases with time after addition of Cu due to a so-called “ageing process” (Oorts et al. 2006a; Tom-Petersen et al. 2004). To take into account the time after contamination in Cu solubilization, kinetic descriptions of Cu availability have emerged (Ma et al. 2006a, b; Zeng et al. 2017). These studies showed that not only the final distribution of Cu but also the kinetics of availability also depend on soil factors. Two different kinetics were identified. One concerns a rapid diffusion of Cu (from minutes to month) mostly controlled by diffusion processes and associated nucleation — precipitation — which will rather depend on soil structure (Ma et al. 2006a; Zeng et al. 2017). The second concerns a slow diffusion of Cu (months to decades) also controlled by the temperature, the pH and soil OM with a faster decrease in availability (e.g. less Cu in soil solution) at higher pH or higher OM (Ma et al. 2006b; Zeng et al. 2017). The soils with low OM content or low pH are hence not only the more prone to exhibit the highest (bio)-available Cu, but they are also the more prone to exhibit it longer (months rather than days or week) after contamination. Thus, in the case of regular Cu input the (bio-)available Cu amount might be higher than estimated in the present study due to non-equilibrium after contamination.

## Conclusion

In this study, we reviewed the empirical equations to estimate available and bio-available Cu from soil total Cu and pedological factors currently measured. On the basis of 29 equations, our results emphasize the dependence of available Cu to pH but also that bio-available Cu is much more dependent on pH than available Cu. The application of the equations at the European scale highlighted similarities as well as differences between areas of risks regarding three different metrics. Areas with a high level of total Cu and high risks of available Cu were more similar than those with bio-available Cu. Indeed, around 74% of the grid points exhibited comparable risks in term of either total or available Cu against 31.5% of the grid points exhibiting comparable risks in term of either total or bio-available Cu. Besides, at the European scale, some regions that classified without risk regarding their total Cu concentration may in turn be considered at risk considering available Cu or considering bio-available Cu. Our computational results show that about 20% of the grid points may be concerned by an underestimation of risk regarding total Cu against available Cu and 39% may be concerned by an underestimation of risk regarding total Cu against bio-available Cu. These areas are non-negligible and underlined the need to estimate local risks beyond the total Cu soil content, with regard to a specific effect in our case biological availability or environmental availability.

## Supplementary Information

Below is the link to the electronic supplementary material.

Supplementary file1 (DOCX 3293 KB)

## Data Availability

The datasets generated and analyzed during the current study are available in the DataINRAe repository, https://data.inrae.fr/dataset.xhtml?persistentId=10.15454/OWI8JR
