# Supplementary material for: Mapping risks associated with soil copper contamination using availability and bio-availability proxies at the European scale
Source: Environ Sci Pollut Res Int. 2022 Oct 15;30(8):19828–44. doi: 10.1007/s11356-022-23046-0 (PMC9938047; doi:10.1007/s11356-022-23046-0)
Supplement: Supplementary file 1 — Supplementary file1 (DOCX 3293 KB) [file 11356_2022_23046_MOESM1_ESM.docx]

Supplementary table 1 : Deciles of concentration of total Cu (mg.kg soil^-1^ of Cu), Cu in solution (µg L^-1^ of Cu) and pCu (-log(free Cu)) for the outliers of total Cu (total Cu>30 mg.kg soil^-1^ of Cu), Cu in solution (Cu in solution > 10.71 µg L^-1^of Cu) and free Cu (pCu<(-0.45)).

|  | 0% | 1% | 5% | 10% | 15% | 20% | 25% |
| --- | --- | --- | --- | --- | --- | --- | --- |
| Cu total >36.8 mg.kg^-1^ of Cu | | | | |  |  | |
| Cu available | 2.2 | 6.5 | 7.8 | 8.6 | 9.1 | 9.6 | 10.1 |
| Cu bio-available | -1.6 | -0.3 | 0.6 | 0.8 | 1.0 | 1.1 | 1.3 |
| Cu available>10.71 µg.L^-1^ of Cu | | | | |  |  | |
| Cu total | 13.5 | 19.3 | 23.6 | 26.6 | 28.9 | 31.0 | 33.0 |
| Cu bio-available | -1.7 | -0.6 | 0.1 | 0.4 | 0.7 | 0.8 | 1.0 |
| Cu bio-available (pCu)<-0.45 | | | | |  |  | |
| Cu total | 2.2 | 4.1 | 5.6 | 6.5 | 7.0 | 7.6 | 8.1 |
| Cu bio-available | 0.6 | 1.2 | 1.4 | 1.5 | 1.6 | 1.7 | 1.8 |

|  | 40% | 50% | 60% | 70% | 75% | 80% | 85% | 90% | 95% | 99% | 100% |
| --- | --- | --- | --- | --- | --- | --- | --- | --- | --- | --- | --- |
| Cu total >36.8 mg.kg soil^-1^ of Cu | | | | | | | | | | | |
| Cu available | 11.4 | 12.3 | 13.2 | 14.3 | 14.9 | 15.6 | 16.5 | 17.8 | 20.0 | 24.7 | 45.0 |
| Cu bio-available | 1.6 | 1.8 | 2.0 | 2.1 | 2.2 | 2.3 | 2.4 | 2.5 | 2.6 | 2.8 | 3.3 |
| Cu available>10.71 µg.L^-1^ of Cu | | | | | | | | | | | |
| Cu total | 37.8 | 40.7 | 43.6 | 47.0 | 49.0 | 51.3 | 54.0 | 57.8 | 64.4 | 79.2 | 128.2 |
| Cu bio-available | 1.5 | 1.7 | 1.9 | 2.1 | 2.2 | 2.3 | 2.4 | 2.5 | 2.6 | 2.8 | 3.1 |
| Cu bio-available (pCu)<-0.45 | | | | | | | | | | | |
| Cu total | 9.5 | 10.5 | 11.7 | 13.5 | 14.6 | 15.9 | 17.7 | 20.3 | 24.2 | 35.3 | 90.3 |
| Cu bio-available | 2.1 | 2.3 | 2.7 | 3.5 | 4.0 | 4.6 | 5.2 | 6.1 | 7.8 | 12.3 | 35.6 |

Supplementary table 2 : Percentiles for the difference to the median of total Cu (in % of the median value)

| decile | 0% | 1% | 5% | 10% | 15% | 20% | 25% | 40% | 50% |
| --- | --- | --- | --- | --- | --- | --- | --- | --- | --- |
| percentage of variation with median | -93.9 | -74.0 | -61.9 | -52.7 | -45.3 | -38.8 | -32.7 | -14.1 | 0.0 |

| decile | 60% | 70% | 75% | 80% | 85% | 90% | 95% | 99% | 100% |
| --- | --- | --- | --- | --- | --- | --- | --- | --- | --- |
| percentage of variation with median | 16.2 | 35.8 | 48.3 | 63.9 | 83.9 | 114.8 | 178.6 | 296.7 | 883.6 |

Supplementary table 3: Percentiles for the difference to the median of Cu in solution (in % of the median value)

| decile | 0% | 1% | 5% | 10% | 15% | 20% | 25% | 40% | 50% |
| --- | --- | --- | --- | --- | --- | --- | --- | --- | --- |
| percentage of variation with median | -94.06 | -79.87 | -69.84 | -62.64 | -56.86 | -51.39 | -45.63 | -21.69 | 0.00 |

| decile | 60% | 70% | 75% | 80% | 85% | 90% | 95% | 99% | 100% |
| --- | --- | --- | --- | --- | --- | --- | --- | --- | --- |
| percentage of variation with median | 24.16 | 51.33 | 67.97 | 88.41 | 115.01 | 152.81 | 218.88 | 364.60 | 1241.25 |

Supplementary table 4: Percentiles for the difference to the median of free Cu (in % of the median value)

| decile | 0% | 1% | 5% | 10% | 15% | 20% | 25% | 40% | 50% |
| --- | --- | --- | --- | --- | --- | --- | --- | --- | --- |
| percentage of variation with median | -99.94 | -99.56 | -98.42 | -96.57 | -93.84 | -89.60 | -83.19 | -48.23 | 0.00 |

| decile | 60% | 70% | 75% | 80% | 85% | 90% | 95% | 99% | 100% |
| --- | --- | --- | --- | --- | --- | --- | --- | --- | --- |
| percentage of variation with median | 96.87 | 286.23 | 413.11 | 564.47 | 759.43 | 1048.60 | 1613.57 | 3520.78 | 61678.97 |


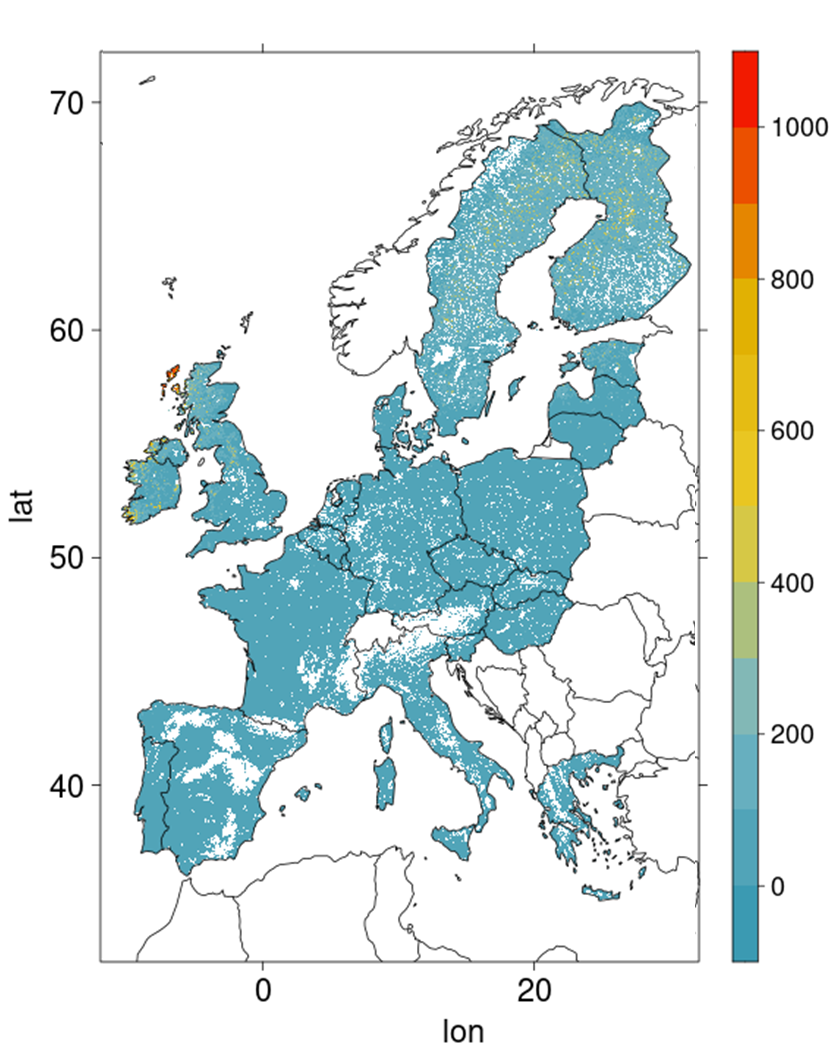


**Supplementary Fig. 1** : Corga map of Europe from https://esdac.jrc.ec.europa.eu/content/topsoil-soil-organic-carbon-lucas-eu25 (de Brogniez et al., 2015).


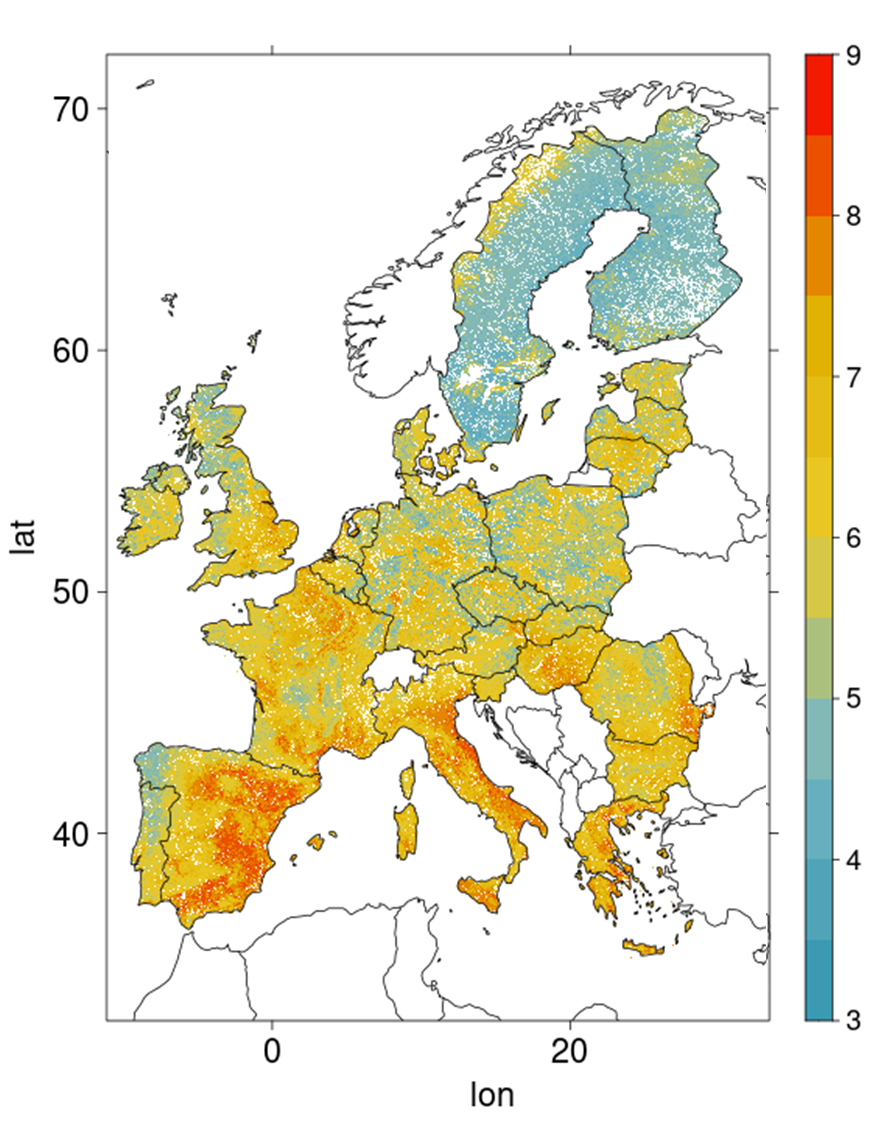


**Supplementary Fig.2** : pH map of Europe from https://esdac.jrc.ec.europa.eu/content/chemical-properties-european-scale-based-lucas-topsoil-data (Ballabio et al., 2019),


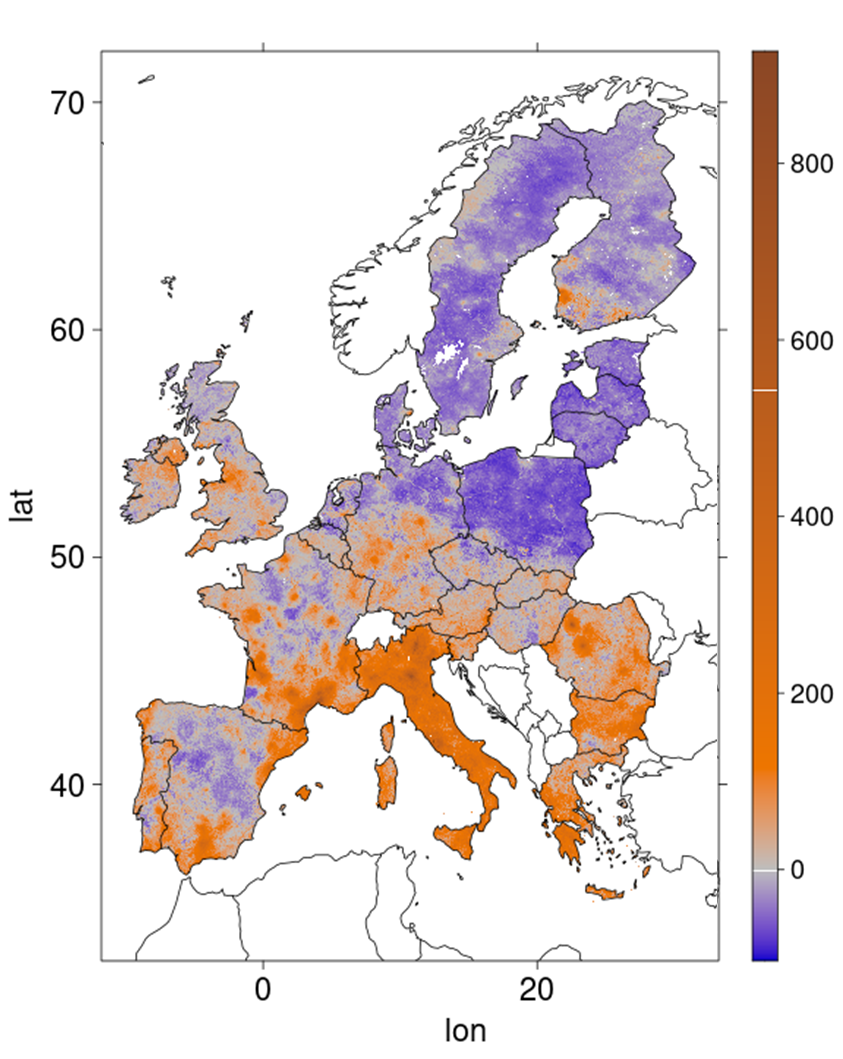


**Supplementary Fig 3** : RI for total Cu value (with median at 13.2 mg.kg soil ^-1^ of Cu) in Europe


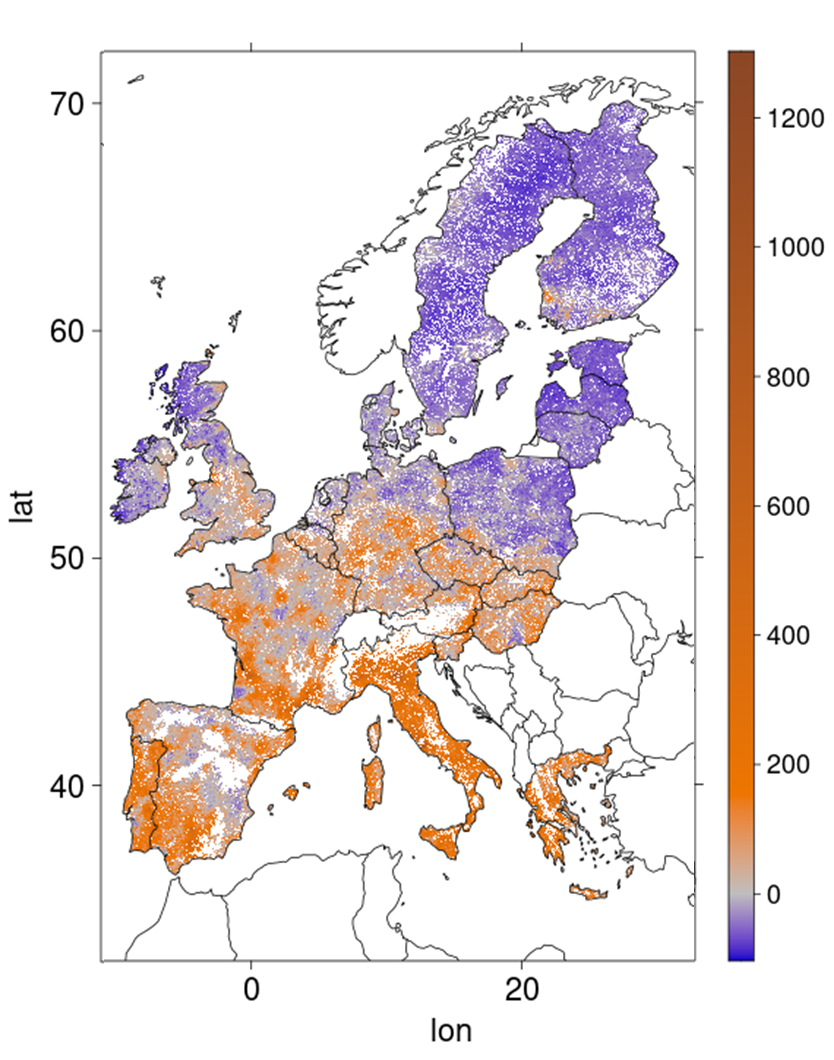


**Supplementary Fig. 4**: RI for available Cu (estimated from eq. 3b) and median Cu in solution (3.36 µg.L^-1^ of Cu) value in Europe


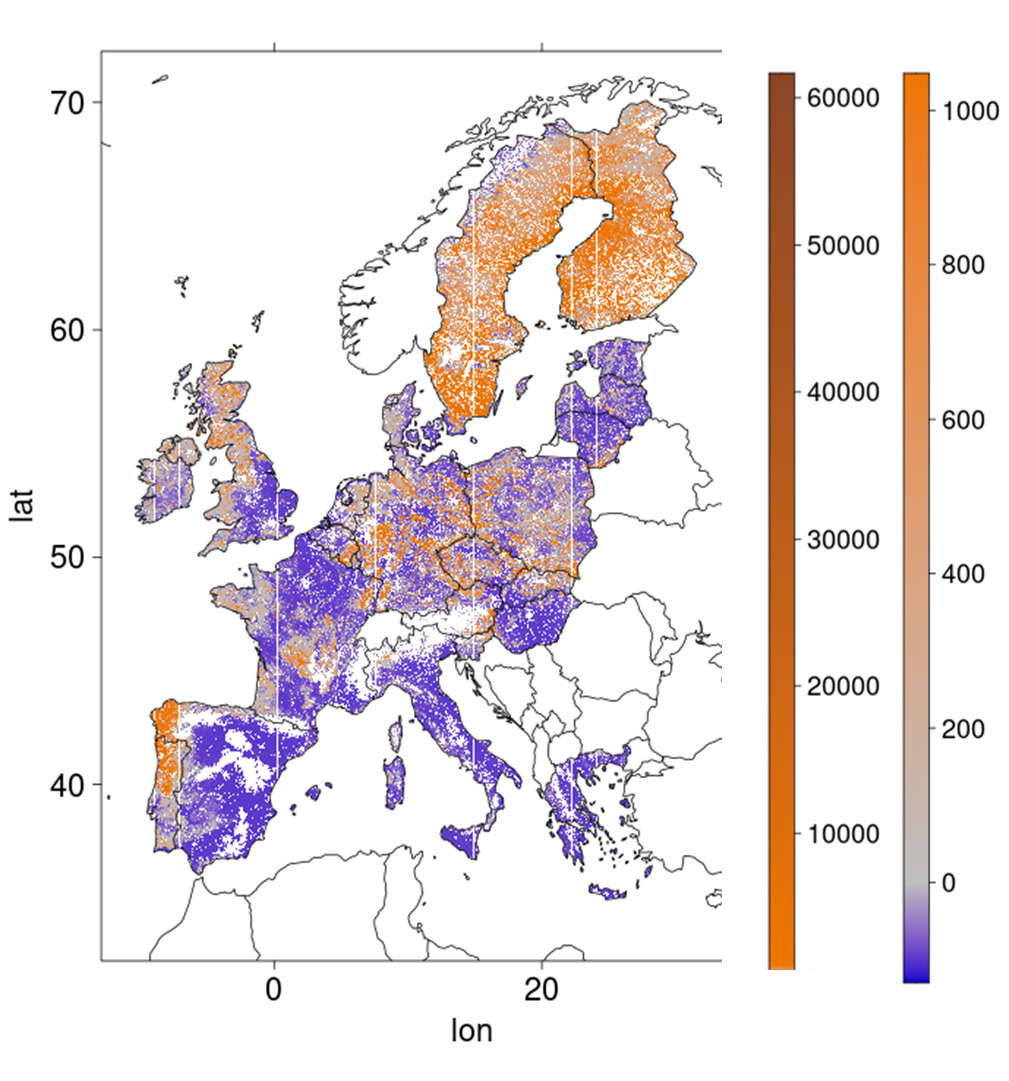


**Supplementary Fig. 5**: RI for bioavailable Cu (estimated from eq 14b.) and median free Cu (0.165 µg.L^-1^ of Cu) value in Europe
